# Chromogranin a: Its Known and Possible Roles in Obstetrics and Gynecology

**DOI:** 10.34763/devperiodmed.20182204.297300

**Published:** 2019-01-14

**Authors:** Michał Niezgoda, Irena Kasacka

**Affiliations:** 1 Department of Histology and Cytophysiology, Medical University of Białystok, Białystok, Poland

**Keywords:** Chromogranin A, endocrine diseases, gynecology, hormone, obstetrics (MeSH-based)

## Abstract

Chromogranin A (CgA) is a prohormone initially extracted from the adrenal medulla, however, increased quantities of CgA are secreted by a wide array of human tissues in the course of a variety of disorders. This protein exhibits a number of interesting endocrine and non-endocrine functions. Here we briefly review the possible involvements of CgA in the areas covered by obstetrics and gynecology. Our account indicates the need to verify its association with the intrapartum fetal stress, and the involvement of CgA in the pathomechanisms of obstetric disorders related to placental dysfunction, as well as in the pathogenesis of endometrioid endometrial cancer as a hormonally regulated malignancy.

## Introduction

When a group of scientists, Blaschko et al., for the first time isolated a group of proteins from the adrenal medulla and labelled them granins over 50 years ago [1], they probably did not anticipate that two decades later another team of researchers would categorize them into 3 separate subtypes: chromogranin A (CgA), chromogranin B (or, secretogranin I), and secretogranin II [2, 3]. Chromogranins have a widespread distribution in human and animal polypeptide hormone producing tissues, including adrenal medulla chromaffin cells, parathyroid chief cells, thyroid parafollicular C cells, pancreatic islet cells, gut enteroendocrine cells, and anterior pituitary cells [4, 5, 6]. They are believed to play a role in hormone packaging within intracellular secretory granules, in hormone secretion, and serve as prohormones for a variety of proteolytic cleavage products [5]. A widely recognized role of CgA is that of a marker for neuroendocrine tumors, also known as NETs. However, at present it is acknowledged that increased quantities of CgA are produced and secreted by a wide array of human tissues in the course of various disorders [7].

Consequently, the aim of this brief review is to indicate a number of the possible involvements of CgA in the mechanisms of health and disease which are of concern to obstetrics and gynecology.

## Chromogranin a and derived compounds

This glycoprotein with a molecular weight of 49 kDa is composed of 439 amino acids [3]. The gene coding for the particle is localized on chromosome 14. CgA belongs to the family of granins, or acidic glycoproteins universally present in all the secretory granules of the cells included in the diffuse neuroendocrine system, or DNES [3].

CgA functions are not fully understood yet. However, some intracellular and extracellular functions of CgA have been elucidated. The intracellular functions comprise the initiation and regulation of dense-core granule biogenesis and sequestration of hormones synthesized by given cells at the *trans*-Golgi network. Interestingly, CgA is co-stored and co-released by exocytosis with secreted hormones [8]. The extracellular functions of the prohormone include the generation of bioactive peptides of endocrine nature. Specifically, depending on where a particular sequence of amino acids was interrupted, CgA can break down into a number of active substances, such as vasostatin, chromostatin, and pancreastatin (Table I) [8, 9]. The converting enzymes of the prohormone are cathepsin L, plasmin, and kallikrein [8]. The resultant peptides exert their actions locally, mostly in an autocrine and paracrine manner [9].

**Table I:** Derivatives resulting from the selective decomposition of chromogranin A (CgA) into particular fragments and their major functions and roles. Adapted from [8] and [9]. PTH - parathormone, DCG - Dense-core secretory granule, nAchR - nicotinic acetylcholine receptor.

Protein/peptide (abbreviation)	Amino acid sequence in CgA	Principal functions/properties ascribed
CgA	1-439	DCG biogenesis and hormones sequestration at the Golgi level
Vasostatin I (VST I)	1-76	vasodilating, antiadrenergic, antiangiogenic, and proapoptotic properties; inhibits PTH secretion, promotes cell adhesion; inhibits endothelial cell proliferation/migration; antimicrobial properties
Vasostatin II (VST II)	1-115	antimicrobial, vasodilating
Chromacin	176-197	antimicrobial (both bacteriolytic and antifungal)
Pancreastatin (PST)	250-301	inhibits insulin release and glucose uptake, inhibits PTH release, inhibits glycogenolysis, stimulates glucagon and histamine release
Catestatin (CST)	352-372	inhibits nAchRs and catecholamine release, vasodilating properties; induces endothelial cell proliferation/migration; reduces cardiac contractility
Serpinin, or serine protease inhibitor	402-439	DCG biogenesis; proadrenergic properties; cell death inhibitor

As it is shown in Table 1, several of the CgA-derived peptides present with opposing counter-regulatory effects, for example vasostatin I and catestatin are antiadrenergic, whereas serpinin is proadrenergic. Vasostatin I inhibits endothelial cell proliferation and migration while catestatin supports these phenomena. Vasostatin I demonstrates proapoptotic properties and serpinin is a cell death inhibitor, etc. Thus, tissue-specific modifications of the CgA chain length result in different bioactive compounds that warrant varied, possibly also tissue-specific physiological effects. It is useful to keep in mind that, as a negative regulator, pancreastatin causes e.g.: insulin resistance due to its inhibitory effect on glucose-stimulated insulin secretion, inhibition of glucose uptake by various cells, and inhibition of lipogenesis in adipocytes [8, 10]. In contrast, catestatin mediates direct vasodilation by histamine-induced production of nitric oxide and is a potent endogenous inhibitor of catecholamine secretion and of catecholamine-mediated arterial hypertension. Another beneficial effect of catestatin is that it decreases obesity by both promoting lipid flux from the adipose tissue (or, lipolysis) and enhancing leptin receptor signaling [8].

It is of interest and in line with the principle of CgA’s co-storage and co-release with secreted endocrine regulatory substances [8] that the prohormone is a major cargo in insulin secretory vesicles within pancreatic β-cells, where it is processed mainly to vasostatin- and catestatin-containing fragments [11]. A recent review indicated a new role of CgA as a potential marker for diabetes [12].

## Chromogranin a and pregnancy

During pregnancy, intrauterine tissues express and synthesize CgA mRNA and protein and secrete it into the biologic fluids of pregnancy [13]. Consequently, during pregnancy new sources of CgA are present, such as placental tissue [14], and hence CgA concentrations in the maternal and fetal blood may be subject to modification. There have been several efforts to explore this.

**Fig. 1:**
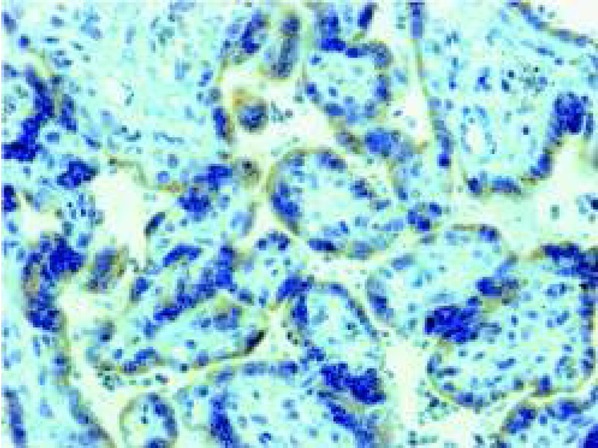
Photomicrograph of human placenta after immunohistochemical staining of chromogranin A (CgA) in the syncytiotrophoblast villi using ‘Monoclonal Mouse Anti-Human Chromogranin A Clone DAK-A3’ antibody from DAKO (Glostrup, Denmark; Catalog number: MO869). Magnification 200 x.

In one study, maternal blood CgA concentrations were found not to change significantly throughout pregnancy, whereas the activity of pancreastatin, as studied by its immunoreactivity, did increase as pregnancy advanced [14]. Maternal salivary CgA concentrations increase in the second and the early third trimesters to decrease thereafter [15]. Interestingly, CgA was confirmed to be present in the amniotic fluid, decidual cells, and in trophoblasts. A comparison of double immunofluorescence results from term placentas demonstrated that there is a remarkable colocalization of CgA and human placental lactogen and human chorionic gonadotropin in trophoblast cells. Since the latter two hormones are synthesized by syncytiotrophoblasts, the interpretation of such an outcome was that CgA should specifically be a product of syncytiotrophoblasts [14]. For us, this observation is another interesting example of CgA’s co-storage and co-release with other secreted hormones. Furthermore, Figure 1 presents an image from the archive of the Department of Histology and Cytophysiology, Medical University of Białystok, demonstrating that, indeed, human syncytiotrophoblast cells do positively stain for CgA.

Italian researchers looked at CgA levels during parturition, either via elective Cesarean section, or spontaneous vaginal delivery, at term. CgA levels were highest in umbilical cord blood; umbilical cord plasma and amniotic fluid CgA levels were significantly higher at vaginal delivery than at C-sections, while maternal plasma levels remained virtually unchanged throughout labor and a 2-hour postpartum period [13]. Also a French study found that CgA and noradrenaline plasma concentrations were significantly higher in infants born by vaginal delivery than by elective C-sections. It is of note that no particular difference was observed for adrenaline concentrations. Since a significant correlation was found between CgA and noradrenaline levels, an elegant suggestion arises from this investigation: in the human fetus, the co-release of CgA with stress is connected with noradrenaline and not with adrenaline secretory granules [16]. Furthermore, the combined data support an association of CgA with the intrapartum fetal stress during vaginal delivery. Somewhat in line, Belgian authors found a trend associating the CgA concentration in the umbilical cord blood sampled at birth with a history of maternal smoking [17].

These data suggest that it is justified to further explore the associations of CgA with intrapartum fetal stress and such disorders of placental dysfunction, as preeclampsia [18], intrauterine growth restriction [19], or early pregnancy loss [20], to name a few.

## Gynecologic involvements

When studied histologically, both CgA and secretogranin II are commonly found to be associated with luteinizing hormone (LH) and/or follicle-stimulating hormone within specialized secretory granules in pituitary gonadotropic cells and, therefore, it is anticipated that they play an important role in the differential secretion of gonadotropins. Studies in rats before and after ovariectomy demonstrated that estrogens decrease anterior pituitary CgA mRNA, whereas ovariectomy increases CgA mRNA levels [5]. Thus, estrogens are one factor negatively influencing the CgA biosynthesis. In contrast, as studied in a mouse pituitary gonadotropic cell line, adrenal corticosteroids (dexamethasone in particular) exert a positive effect on pituitary CgA, in a similar way as the gonadotropin-releasing hormone from the hypothalamus [21]. Secretoneurin, which is a functional secretogranin II-derived peptide, was shown to stimulate the production and release of LH in this cell model [22], possibly by facilitating LH trafficking into secretory granules [21].

Interestingly, women of reproductive age with a high degree of premenstrual psychoemotional symptoms have increased salivary CgA concentrations in the late-luteal phase [23]. Moreover, we would like to draw attention to the need for verification of the involvement of both CgA and pancreastatin in the pathomechanism(s) of endometrioid endometrial cancer. Two serious arguments support this suggestion. Over the past decades, there has been a tremendous increase in the incidence of overweight and obesity worldwide, which are established risk factors for this most prevalent gynecological malignancy nowadays. As indicated above, pancreastatin causes insulin resistance. Second, a proportion of endometrioid adenocarcinomas demonstrate neuroendocrine features of which CgA is an established marker. Initial studies of this topic [24] clearly require further exploration.

## Conclusions

The presented account of the possible roles and involvements of CgA in obstetrics and gynecology warrants further interest in launching research on the prohormone’s clinical significance, apart from its application as a NETs marker. In particular, our account indicates the need to verify its association with intrapartum fetal stress and the involvement in the pathomechanisms of obstetric disorders related to placental dysfunction, as well as in the pathogenesis of endometrioid endometrial cancer as a hormonally regulated malignancy.
